# Long-term outcomes of COVID-19 intensive care unit survivors and their family members: a one year follow-up prospective study

**DOI:** 10.3389/fpubh.2023.1236990

**Published:** 2023-08-08

**Authors:** Raphael Laurent, Patricia Correia, Raphael Lachand, Eric Diconne, Eric Ezingeard, Franklin Bruna, Pierre-Alban Guenier, Dominique Page, Sophie Périnel-Ragey, Guillaume Thiéry

**Affiliations:** ^1^Service de Médecine Intensive Réanimation G, Centre Hospitalier Universitaire de Saint-Etienne, St Etienne, France; ^2^Université Jean Monnet, Saint-Etienne, France; ^3^Research on Healthcare Performance RESHAPE, INSERM U1290, Université Claude Bernard Lyon, Lyon, France; ^4^Laboratoire INSERM 1059 SAINBIOSE, Université Jean Monnet, Saint-Etienne, France

**Keywords:** COVID-19, ARDS, intensive care, post-traumatic stress disorder, follow-up, mental disorders, family members

## Abstract

**Purpose:**

To describe the long-term physical, functional and mental status of COVID-19 intensive care unit (ICU) patients and their family members 1 year after ICU discharge.

**Methods:**

We performed a prospective observational cohort study among patients admitted to the ICU for COVID-19-associated respiratory failure and their family members. Patients attended a one-year follow-up consultation with family members. Physical, functional and respiratory outcomes were collected. In addition, participants completed the Hospital Anxiety and Depression Scale and the Revised Impact of Event Scale. Qualitative components were collected during a 2-h face-to-face interview.

**Results:**

Fifty-four patients and 42 family members were included. Thirty-four (63%) patients reported chronic fatigue and 37 (68.5%) dyspnea. Computed tomography scans were abnormal in 34 patients (72.3%). Anxiety symptoms were present in 23 (48%) patients and 26 (66%) family members, depression in 11 (23%) and 13 (33%), and post-traumatic stress disorder in 12 (25%) and 23 (55%), respectively. Visit limitation was reported as the most painful experience for family members. Numerous patients recalled nightmares that contributed to the anxiety. Long-term reconstruction was difficult for both patients and family members.

**Conclusion:**

The vast majority of patients and their relatives reported long-term consequences on various physical and mental components, leading to a profound impact on their well-being.

## 1. Introduction

Patients who survive after severe respiratory failure and prolonged intensive care unit (ICU) stay may experience long-term morbidities related to the critical illness, ICU treatment or the ICU environment (1–3). Apart from the respiratory sequelae of the lung injury itself and mechanical ventilation, ICU survivors are at risk of functional impairment, depression, anxiety and post-traumatic stress disorder (PTSD) that may profoundly affect their quality of life (1, 4–6). Beyond the patients, family members may be also affected by the ICU stay and suffer from anxiety, depression and PTSD (1, 7).

The emergence of coronavirus disease 2019 (COVID-19) rapidly evolved into a worldwide pandemic and a high number of patients required admission in ICUs due to severe acute respiratory failure associated with invasive treatments, such as mechanical ventilation and prolonged ICU stay (8). Short-term follow-up studies have reported a high prevalence of physical, mental or cognitive symptoms and a wide range of respiratory sequelae in ICU survivors (9–13). More recently, some authors have reported similar findings in patients with longer follow-up (14, 15). Similarly, family members have described the traumatic experience of ICU stay, irrespective of whether the patients survived or not (16–18). As severe COVID-19 and ICU stay may have significant consequences on the family unit, we aimed to evaluate concomitantly the overall one-year outcomes of ICU survivors and their family members in their physical and psychological dimensions.

## 2. Methods

### 2.1. Patients and study setting

This was a prospective observational cohort study conducted in the 20-bed medical ICU of the Saint-Etienne University Hospital (France). The study is reported in accordance with STrenghtening the Reporting of Observational studies in Epidemiology (STROBE) recommendations (19). During the first (March 2020) and second waves (October 2020) of the pandemic, our ICU was entirely dedicated to COVID-19 patients. Patients were treated by the usual team of highly trained physicians and nurses. Due to the surge and the need to activate more ICU beds, additional physicians and nurses were recruited to reinforce the team. On March 17 2020, visitor access to the ICU was prohibited, with exceptions for end-of-life patients. From April 10, visits were allowed again, but restricted to one person per day for a maximum of 2 h. Progressively, the visitation policy was made more flexible and two visitors per day with no limitation on duration were allowed until the end of the study period. In order to replace the ICU diary, which could not be technically used because of visit restrictions, family members were encouraged to send emails and pictures that were included in a notebook by the team and given to the patient at ICU discharge.

When necessary, patients were transferred to a rehabilitation unit after discharge from the ICU or the hospital. The rehabilitation program is focused on physical rehabilitation and does not include a psychological follow-up. However, while in the ICU, the ICU psychologist identified patients and family members at risk of psychological troubles and proposed an appointment.

As part of routine care in our ICU, a one-year follow-up consultation is proposed at discharge to all ICU survivors with a stay of 7 days or more and their family members, irrespective if they were intubated or not. Patients were included in the study if they were eligible for the one-year post-ICU consultation and if they had been admitted to the ICU for severe COVID-19. Exclusion criteria were patients hospitalized in the ICU for less than 7 days, aged <18 years, or hospitalized in the ICU for COVID-19 without respiratory failure.

### 2.2. Follow-up protocol

All patients hospitalized in the ICU for COVID-19-associated respiratory failure from 3 March 2020 to 20 December 2021 with a length of stay of 7 days or more were invited to attend a follow-up consultation. Two months prior to the date of the consultation, patients received a notification by mail with a scheduled appointment and an explanatory cover letter. Also included was a prescription for a low-dose thoracic computed tomography (CT) scan and laboratory tests, including an ionogram, blood urea and creatinine levels, a hemogram and albumin concentration, as well as self-evaluation questionnaires for themselves and their closest relative. Patients were free to decline or reschedule the consultation by contacting the ICU secretary.

All consultations were performed by a senior intensive care physician and at least one member of the nursing staff (nurse, psychologist, physiotherapist, auxiliary nurse). A psychologist participated in the consultations for some patients, especially those in who were identified or suspected to have psychological troubles before the consultation, or for those who were in contact with the psychologist during the ICU stay. Each consultation was scheduled for 2 h and divided into four parts. First, the medical history, including the ICU stay and post-ICU period, was reviewed with the patient and a family member and additional information was given by the physician or nurse at the patient’s request. Second, the patient’s psychological status was assessed through a free talk and analysis of the questionnaires. If necessary, the patient was referred to a psychologist or a psychiatrist. Third, a physical examination was performed, including resting oximetry, cardiopulmonary auscultation, blood pressure and weight measurements. The CT scan and laboratory tests were interpreted and the results given to the patient. When required, the patient was referred to a general practitioner or a specialist. Four, the family member was interviewed, following by an analysis of his/her questionnaire. If possible, part of this interview was conducted without the presence of the patient. When needed, the family member was referred to a psychologist or a psychiatrist. Finally, at the end of the consultation, a visit to an ICU room was proposed to the patient.

### 2.3. Data collection

Demographic characteristics and clinical data of the ICU stay were collected for each patient. During the consultation, patient data related to physical, mental, and functional outcomes were also collected, as well as perceptions of ICU discomfort and mental health outcomes of family members. In addition, patients and family members were encouraged to share their feelings through a semi-directive interview focused on three questions: Did this ICU experience change your life on a daily basis? Will it have an influence on your future? Was it difficult to come back to the hospital?

### 2.4. Outcome measures

Physical symptoms were collected through the physical examination. Baseline patient characteristics, comorbidities and clinical data were retrieved from the patient’s electronic health record. Severity of the acute illness was assessed by the Simplified Acute Physiology Score (SAPS 2) measured within the first 24 h of the ICU stay (20). Respiratory status was evaluated by the Modified Medical Research Council Dyspnea Scale (mMRC) scale and pulse oximetry was measured at rest.

Self-reported quality of life was measured by the Short-Form General Health Survey (SF-12) (21), which comprises 12 items from the Medical Outcomes Study 36-Item Short-Form Health Survey (SF-36) and provides a Physical Component Summary (PCS-12) and Mental Component Summary (MCS-12) score. Both are measured on a 0 to 100 scale where higher scores represent a better perceived quality of life.

The patient’s mental health status was assessed by the Hospital Anxiety and Depression scale (HADS) and the Revised Impact of Event Scale (IES-R). The level of anxiety and depression was measured with the HADS scale (22), which allocates each patient to one of three categories, based on the individual final scores. A score > 11 indicates a confirmed case and a score > 7 in each domain indicates a moderate or strong probability of anxiety or depression, respectively. PTSD was measured by the IES-R (23, 24). A score > 20 indicates reactions of clinical importance and a score > 33 indicates a high probability of a PTSD diagnosis. A total mean score > 1.6 suggests symptom levels compatible with a diagnosis of PTSD (24).

Assessment of perceived discomfort during ICU stay was measured by the *French* self-reported *discomfort* IPREA questionnaire (Inconforts des Patients de REAnimation questionnaire) (25). This questionnaire provides measurements of perceived discomfort such as noise, excessive light, bed-related discomfort, sleep deprivation, thirst, hunger, overall discomfort related to mechanical ventilation, pain, being tied down by perfusion lines, connecting wires, and cables, no respect for intimacy, anxiety, isolation, limited visiting hours, absence of a telephone, and lack of information. Patients were asked to complete the 16 items of the questionnaire and rate the severity of each discomfort source experienced during the ICU stay on a 10-point scale, with 0 representing no discomfort and 10 representing the worst discomfort ever perceived.

Open questions were added to the questionnaires in order to evaluate the patient’s perception of the ICU stay, their feelings about recovery and the return to family, social and professional life. During the 2-h consultation, an open discussion took place and patients and family members were asked about three domains: potential painful experiences during the ICU stay; impact of the ICU stay on the family unit; and impact of the ICU stay on their perception of the future. Striking quotes were collected by the physician or the nurse in charge leading the consultation.

### 2.5. Statistical analysis

Continuous variables were presented as the mean with standard deviation (SD) or medians with interquartile ranges (IQR 25–75), depending on their distribution. Categorical variables were presented as proportions. For analyses using questionnaires such as the HADS and IES-R, scores were analyzed as dichotomous, according to their respective cutoffs. A multivariate analysis was performed using a logistic model to identify factors involved in the variability of mental disorders. Mental disorders were defined by a HADS score > 10 for anxiety, a HADS score > 10 for depression or an IES-R score > 33. The first group of variables explored demographic characteristics and acute conditions: age, gender, SAPS2, length of ICU stay, length of hospital stay, renal replacement therapy, invasive mechanical ventilation, duration of invasive mechanical ventilation, ventilator-associated pneumonia and shock. The second group of variables explored the long-term physical outcomes: physical activity, dyspnea, mMRC scale, chronic fatigue, sleep disorders and abnormal chest CT-scan. The final model was chosen using Stepwise Akaike Information Criterion backward elimination using the “step” function of R (version 4.3.1).

## 3. Results

### 3.1. Patient demographic and clinical characteristics

**Figure 1:**
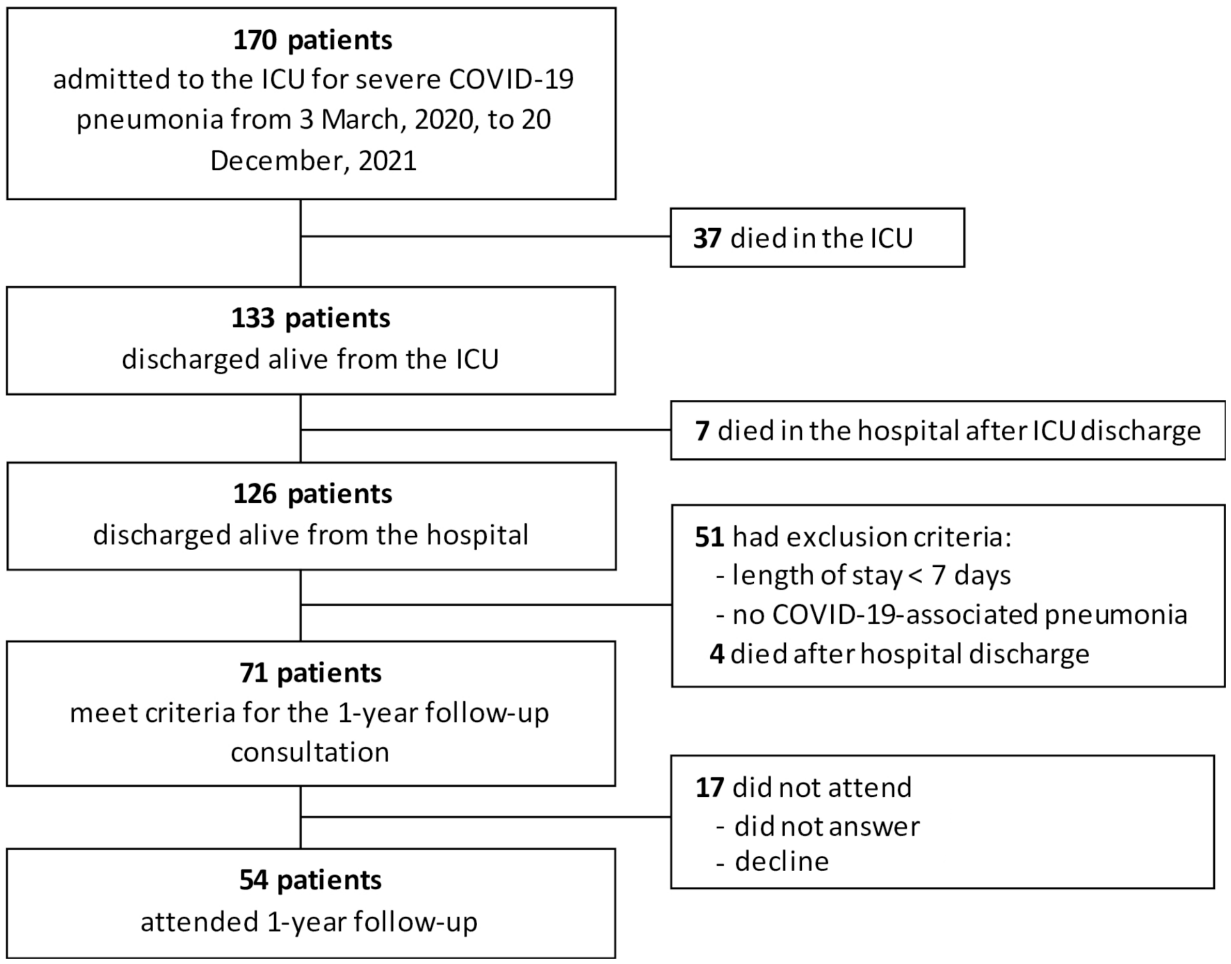
Study flowchart.

From March 3 2020, to December 20 2020, 170 patients were hospitalized for severe COVID-19-associated pneumonia. ICU and hospital mortality rates were 22 and 25%, respectively. Of the 126 patients who were discharged alive from the hospital, 71 met the criteria for the one-year follow-up consultation. Fifty-one patients had a length of stay < 7 days or no COVID-19-associated pneumonia and four died after hospital discharge. Seventeen patients declined or did not respond. Finally, a total of 54 patients attended the consultation and were included in the study (Figure 1). Of the 54 patients in the cohort, 31 were transferred to a rehabilitation center after discharge from the ICU. Questionnaires were completed by 48 of 54 patients.

Median age was 66 (IQR, 58–72.5) years; 83% were men. The most common comorbidities were hypertension (57%) and obesity (39%). Most patients (77%) were intubated for a median duration of mechanical ventilation of 14 (IQR, 7–19) days. Patients who did not require intubation were all treated with high flow nasal oxygen. One patient required extracorporeal membrane oxygenation. Median ICU and hospital length of stay were 19 (IQR, 12–26) and 30 (IQR, 23–40) days, respectively. For 31 patients who were transferred to a rehabilitation unit, the total length of stay was 50 (IQR, 29–63) days. Patient characteristics are presented in Table 1. None of these patients were vaccinated because they were all hospitalized during spring and fall of 2020, before vaccines were available. Nine patients were immunocompromised (i.e., solid tumors, hematological malignancies, immunosuppressive drugs). Delirium was observed in 17 patients. All of them were mechanically ventilated and delirium occurred during or after weaning from mechanical ventilation. Twenty-two patients received corticosteroids, all were hospitalized after the results of the Recovery trial were published (26).

**Table 1:** Demographic and clinical characteristics of patients with COVID-19 treated in the intensive care unit (*n* = 54).

Characteristic demographic and clinical characteristics of patients	*N* (%)
Age (years)	
Median (interquartile range)	66 (58–72.5)
Male (%)	45 (83%)
Comorbidities—no (%)	
Hypertension	31 (57)
Obesity (body mass index > 30 at ICU admission)	22 (40)
Diabetes mellitus	16 (29)
Cardiovascular disease	18 (33)
Chronic respiratory disease	10 (18)
Chronic kidney disease	1
Patients with at least one comorbidity	48 (89)
SAPS 2* at enrolment	
Median (interquartile range)	37 (28.5–45.25)
Acute conditions—no (%)	
Cardiovascular dysfunction	9 (16)
Renal dysfunction	4 (7)
Ventilator-associated pneumonia	33 (61)
Patients with at least one of these acute conditions	33 (61)
Invasive mechanical ventilation	
No. of patients (%)	42 (77)
No. of days—median (interquartile range)	14 (7–19)
Duration of ICU stay—days	
Median (interquartile range)	19 (12.25–26.75)
Duration of hospital stay—days	
Median (interquartile range)	30 (23–40)
Duration of in-hospital rehabilitation stay—days (*n* = 31)	
Median (interquartile range)	50 (29–63.75)

* SAPS 2: simplified acute physiology score 2.

A total of 42 family members completed the questionnaires and were included in the study. Of these, 28 were present during the follow-up consultation.

### 3.2. Physical outcomes

Thirty-four (63%) patients reported chronic fatigue that affected their daily activities. Most patients had returned to their baseline weight at 1 year. Median body weight was 83 kg (IQR, 75–98) before ICU admission and 81 kg (IQR, 75–97) on the day of consultation, despite a substantial weight loss during ICU stay. Albumin dosage was available in 37 (68%) of 54 patients and had mostly returned to normal values [42.5 g/L (40–43.85)]. One year after ICU discharge, 33 (61%) of 56 patients reported that they resumed their hobbies. One-half (9) of the pre-ICU employed patients had returned to work [4 (22%) part-time; 5 (28%) full time].

### 3.3. Respiratory outcomes

Among the 54 ICU survivors, 37 (68.5%) expressed symptoms of shortness of breath median range according to the mMRC classification was 2 [IQR, 1–2]; 9 (16%) patients had severe dyspnea (grade 3 on the mMRC scale). At one-year follow-up, none required the use of supplemental oxygen, and median pulse oximetry at rest was 96%. Forty-seven patients (82%) underwent low-dose thoracic CT before the follow-up consultation. Chest CT scans were abnormal in 34 (72.3%) patients at 12 months. When present, radiologic changes included bronchiectasis in most cases (42.5%), ground glass opacities (40.4%), reticulations (31.9%) and anecdotically atelectases, nodules and alveolar consolidations.

### 3.4. Mental outcomes in patients and in relatives

**Figure 2:**
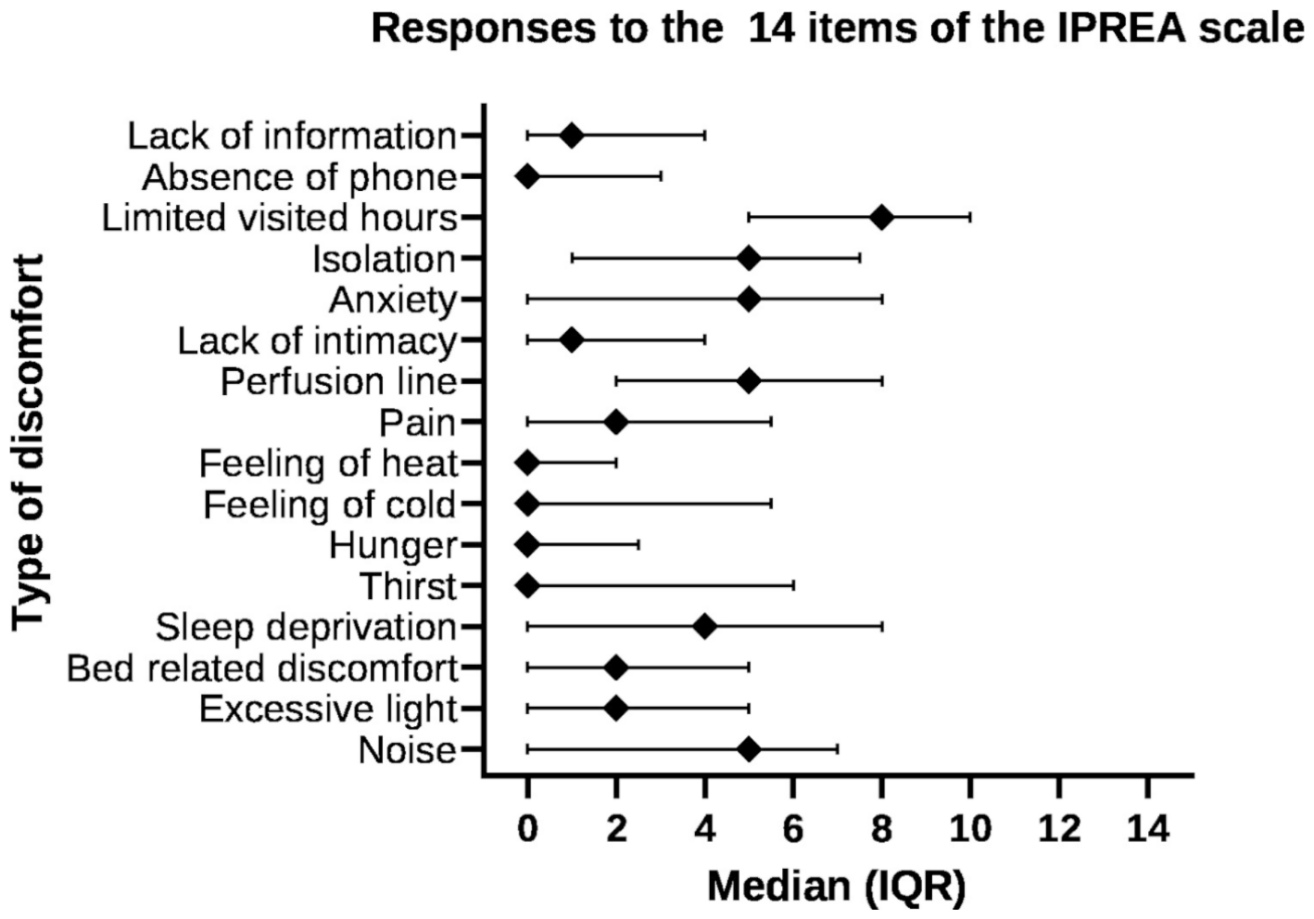
Discomfort IPREA items among the 44 patients responders (median, interquartile range).

Eight patients (16%) had symptoms of anxiety, 6 (12%) had symptoms of depression, and 51% had a high probability of PTSD (whether defined by a score > 10 or by a mean score > 1.6). Among relatives, 17 (44%) had symptoms of anxiety, 8 (21%) had symptoms of depression, and 23 (55%) had a high probability of PTSD (Table 2). In multivariate analysis, the following demographic characteristics were associated with long-term mental disorders: female gender (OR, 26.38 [95%CI, 2.83–668.66]), SAPS 2 (OR, 0.92 [95%CI, 0.84–0.98]) and mechanical ventilation (OR, 100.76 [95%CI, 5.02–9100.73]; Figure 1 ESM). Long-term physical condition associated with long-term mental disorders is mMRC dyspnea scale (OR, 3.29 [95%CI, 1.15–11.47]; Figure 2 ESM).

**Table 2:** Prevalence of symptoms in patients and relatives at one-year following intensive care unit treatment for COVID-19.

	Patients (*n* = 48)	Relatives (*n* = 42)
IES-R scores		
Median (interquartile range)	21 (8–34)	46.5 (19–58)
IES-R ≥ 18, no (%)^§^	24 (51)	31 (71)
IES-R ≥ 33, no (%)^§§^	12 (25)	23 (55)
Mean IES-R ≥ 1.6 (%)^§§§^	12 (25)	
HADS scores		
Anxiety, median (interquartile range)	7 (3–10)	10 (7–14)
Depression, median (interquartile range)	4 (2–7)	5 (2–9)
HADS anxiety ≥ 8, no (%)^*^	23 (48)	26 (66)
HADS anxiety ≥ 11, no (%)^**^	8 (16)	17 (44)
HADS depression ≥ 8, no (%)^*^	11 (23)	13 (33)
HADS depression ≥ 11, no (%)^**^	6 (13)	8 (21)

* A score of 8 or more indicating symptoms of anxiety or depression.

** A score of 11 or greater indicating definite cases.

§ A score of 18 or more indicating reactions of clinical importance.

§§ A score of 33 or more indicating severe symptoms with high a probability of a PTSD diagnosis.

§§§ A mean score of 1.6 or more indicating a high probability of a PTSD diagnosis.

IES-R, revised impact of event scale; HADS, hospital anxiety and depression scale. Percentages may not sum to 100 owing to rounding. Of the 54 patients included, 47 completed the IES-R, 48 completed the HADS. Of the 42 relatives included, 42 completed the IES-R, 39 completed the HADS.

### 3.5. Perceptions of ICU discomfort

Forty-six of 54 patients completed the IPREA questionnaire. The mean overall score of discomfort was 3.3/10 (± 1.8). The highest scores were observed for limited visiting hours (mean 7.0 ± 3.1; median 8 [IQR, 5–10]) and perfusion lines (mean 5.1 ± 3.5; median 5 [IQR, 2–8]). The most frequently perceived discomforts with a score > 5/10 were limited visiting hours (*n* = 31), sleep perfusion lines (*n* = 21), anxiety (*n* = 19), isolation (*n* = 19), noise (*n* = 18), and sleep deprivation (*n* = 17). Other items were associated with a mean score < 5. Restrictions of visiting hours was an important discomfort with 62% (*n* = 28) of responders reporting a score higher than 7 (Figure 2).

### 3.6. Self-perceived quality of life

The SF-12 score was available in 48 patients. Median PCS and MCS component summary scores were 44 (IQR, 32–52) and 45 (IQR, 41–48), respectively.

### 3.7. Patients and family members’ narratives

#### 3.7.1. Painful experiences

The two most painful experiences reported by patients were visit limitations and nightmares. Although most patients and family members understood the reasons of visit limitations, they both expressed a feeling of extreme solitude. For family members, visit limitations were more painful than for patients and associated with a feeling of guilt and anxiety. Telephone calls with the ICU team were perceived either as a moment of relief or a threatening experience. Many patients developed tools to overcome this isolation, such as a diary or family meetings.

One-quarter (*n* = 14) of patients had no memory of their ICU stay and their first memories started in the ward or even in the rehabilitation center. Others (*n* = 6) had confused memories that mixed reality and false perceptions.

We report here some of the remarkable quotes from patients: “*I heard the helicopter and then they took me to send me to Asia*” and “*I had captors and wires put on my body and people were spying on the thoughts in my brain*.” Some patients recall memories of pain (*n* = 3) and others of being tied down (*n* = 3). However, one of the main striking findings in the patient narrative were the frequency and intensity of nightmares (*n* = 13). Patients mostly experienced imminent threats: “*It was like I was drowning and I did not know how to swim*,” or terrifying situations: *“I saw nurses killing people and putting them in a train”; “I was kidnapped and I had to put out the fire to escape”; “I was being captured by terrorists in Afghanistan and some special forces came to rescue me, but they had to kill a lot of people to do so. But even though they came to rescue me, I was still captured and it was endless.”*

#### 3.7.2. Impact on families

Despite the difficulties of the ICU and hospital stays, many family members reported that it had strengthened intrafamilial links and led to a feeling of solidarity within the family. However, the end of hospital stay was not synonymous with a return to a normal life for both patients and family members. Due to the persistent physical limitations and psychological consequences for both patients and family members, life in the family unit remained far from what it was prior to hospitalization.

#### 3.7.3. Impact on the vision of the future

For many patients, attending the follow-up consultation was a way to “close the chapter” of the ICU stay. They were ready to start a new life, more focused on taking care of themselves and their family. However, almost all patients expressed a persistent feeling of frailty and vulnerability within the whole family unit. Many patients reported the fear to be contaminated again and to endure a similar hardship once more.

## 4. Discussion

In this prospective monocentric cohort study, COVID-19 ICU survivors were evaluated 1 year after ICU stay, as well their family members. Our results clearly showed the high burden, both for patients and their families, and revealed that families still experienced the consequences of the disease and ICU stay in a substantial proportion of cases. A reduction of physical activity levels was reported by one-third of patients and high levels of anxiety, depression and PTDS symptoms were found in most patients and were even more pronounced in family members.

The reduction of physical activity in our patients is consistent with the findings of previous studies (9, 15, 27–29). In a Belgian study of COVID-19 ICU patients reported by Rousseau et al., up to 87% had not returned to their previous level of activity at 3 months post-discharge (9). Early after ICU discharge, patients experience muscle weakness, which is correlated to self-reported physical limitations and quality of life (28, 30). Interestingly, this limitation persists over the time and remains 1 year after ICU discharge as observed in our patients (14, 15, 27, 28). In a large Dutch cohort study of patients at one-year follow-up after ICU stay, 56% reported experiencing fatigue as in our study, and two-thirds reported new physical medical problems, including fatigue, pain and weakness (16).

In our series, only one-half of previously-employed individuals returned to work. Among these, one-half were only able to work part-time. Thus, only one-quarter of previously-employed patients returned to their initial position. These results are similar to a Dutch cohort in the same population where 58% of survivors employed before ICU admission reported work-related problems (15). It is likely that this is predominantly the result of acute respiratory distress syndrome (ARDS) and mechanical ventilation non-specific to COVID-19 as similar results were found in non-COVID-19 ICU survivors (4, 31).

Although our study was not specifically designed to investigate respiratory conditions, it is noteworthy that 56% of patients reported persisting dyspnea, including one-quarter reporting severe dyspnea. This is consistent with the recent one-year follow-up study of COVID-19 ICU survivors by Eberst et al. who reported that 27% of patients with an abnormal 6-min walk-test had a walking distance lower than predicted. In addition, 38% of patients had a decrease of 4% or more in SpO_2_ (10). Interestingly, in this study, only 8 of 85 patients had persistent impairment of the diffusion capacity of the lungs for carbon monoxide (DLCO) at 1 year (11%) and, among these, only one was likely to have COVID-19-related DLCO alteration. However, other authors have shown that the severity of the CT scan and DCLO impairment was correlated to the severity of the acute disease (32). CT chest abnormalities in our cohort were found in 72% of patients, which was similar to the report by Eberst et al. and higher than findings reported in COVID-19 non-ICU patients (33). The discrepancy between the degree of pulmonary dysfunction and the observed functional limitation suggests that many non-respiratory parameters are involved, as previously described in ICU survivors treated for non-COVID-19 ARDS (4, 34). As a similar functional limitation may be observed in patients who suffered from mild COVID-19 and who did not require ICU admission or even hospital admission, these symptoms may be included in the post-acute sequelae of severe acute respiratory syndrome coronavirus 2 infection (SARS-CoV-2) or “long” COVID (35, 36). Based on these results, minimizing of acute respiratory failure is a priority. Several strategies can be suggested to achieve this goal, such as avoiding invasive mechanical ventilation when possible by using noninvasive strategies (high-flow nasal oxygen or noninvasive ventilation), shortening the duration of sedation by using short-acting drugs and daily interruption of sedatives, and early rehabilitation and mobilization during the ICU stay.

One significant finding of our study was the high proportion of patients who suffered from symptoms of anxiety, depression and PTSD, and the even higher proportion in their family members. Indeed, one-half of patients and nearly three-quarters of family members had symptoms of mental disorders. These results are consistent with other authors and, despite the fact that there are heterogeneous results among published studies, there is no doubt as to the mental burden of ICU treatment in these patients (9, 11, 14, 16, 37, 38). Moreover, these results were confirmed by the patients’ narratives collected during the 2-h consultation, where they were encouraged to talk about their memories and their perception of ICU stay, as well as current feelings. Of note, family members had a higher prevalence of anxiety, depression and PTDS than patients. Our results differ from other authors. In a French study, Azoulay et al. found a similar prevalence of depression, but a lower prevalence of anxiety (25%) and PTSD (20%) at 90 days (39). Similarly, a lower prevalence of anxiety, depression and PTSD have been reported by Heesakers et al. at 12 months (29, 23 and 20%, respectively) (16). The reason to explain such different results remains unclear. Heesakers et al. found that the only factor associated with mental health symptoms 12 months after ICU admission was the presence of anxiety or depression prior to the hospitalization (16). Several strategies can be suggested to improve patient and family outcomes, such as open visitation policies, family members participation in the care of patients, implementation of diaries in which family members are encouraged to write and share photos, ensuring the quality of communication between ICU staff and family members, and psychological support of patients and family members during and after the ICU stay.

Visit limitations were described as a painful experience for many patients and associated with a strong and devastating feeling of isolation for both patients and family members. Even when visiting restrictions were lightened in our ICU, patients and family members still had to endure restrictions applied in wards and rehabilitation units after ICU discharge. The burden of visit limitation is well illustrated by the IPREA score, i.e., 27 of 44 patients (61%) rated the level of discomfort due to visit restrictions as 8 or more on the 10-point IPREA scale.

The impact of visit limitation was even more strongly reported by family members. It was often described as a “nighmare” and definitely played a role in the high levels of anxiety, stress and PTDS observed in family members. Indeed, similar level of mental health disorders have been previously reported by Heesakers et al. (16). Interestingly, Rose et al. showed that even when virtual visitings were implemented during visit limitation periods, 62% of family members reported severe psychological distress (17). In a qualitative study conducted among family members of COVID-19 patients who died in the ICU during the first wave in France, where many ICUs had restricted visitation policies, Kentish-Barnes et al. showed how detrimental it was to prevent family members from seeing their loved ones (18). In this study, family members reported a feeling of great solitude and difficulty in building a relationship with the ICU team. In a qualitative study conducted in family members of critically ill COVID-19 patients, Digby et al. also demonstrated that family members suffered from both the separation with the patient and the inability to build a close relationship the ICU team (40). Similar observations were made at interviews with family members during our follow-up consultations, with the most prevalent being feelings of loneliness, powerlessness and a certain guilt.

Another theme that emerged from interviews with family members was the importance of families in the reconstruction process after hospital discharge. In this perspective, most patients were pleased to attend the follow-up consultations and, for some, they represented the final point of this ordeal and helped them “turn the page” and reconstruct their lives. During follow-up, a modification in patient health management and/or strategy was considered in 31 cases (55%). These interventions concerned medication reconciliation, including modification or dose adjustment (anticoagulant therapy, antihypertensive therapy, etc.), specialized consultations, such as pneumology or ophthalmology, a vaccine booster against SARS-Cov-2, psychological care or other counseling.

Our study has limitations. First, it is a single center study with a limited number of patients. Some of the psychological outcomes of patients and their family members may be specific to our ICU. Nevertheless, similar types of discomfort or mental health outcomes have been described by other studies in different settings. Second, only ICU survivors and their relatives were included. As a result, the psychological outcomes of family members of deceased patients could not be evaluated, whereas it is known that bereaved family members frequently experience mental health symptoms (18). Third, respiratory follow-up is not fully described as it was not the primary goal of the study. Functional respiratory tests were not performed in each patient and we had insufficient data to report these results. Finally, this is a descriptive study that provides few explanatory data. However, we chose to quote some of the patients’ narratives collected during interviews in order to provide an in-depth understanding of patients and family members’ perceptions.

In conclusion, our findings show the heavy impact of severe COVID-19-related ICU stay on patients and their relatives. Although long-term respiratory consequences were somewhat moderate compared to the severity of the acute disease, there was a major global impact on the lives of patients and their family members and the vast majority reported long-term consequences that affected their daily life activities.

## Data availability statement

The raw data supporting the conclusions of this article will be made available by the authors, without undue reservation.

## Ethics statement

The studies involving human participants were reviewed and approved by IRBN982021/CHUSTE. Written informed consent for participation was not required for this study in accordance with the national legislation and the institutional requirements.

## Author contributions

GT and RLau designed the study and drafted the manuscript. GT performed the statistical analysis. FB, PC, ED, EE, P-AG, RLau, RLac, DP, SP-R, and GT included patients. All authors revised the manuscript for important intellectual content, approved the final version, and approved its submission for publication.

## Conflict of interest

The authors declare that the research was conducted in the absence of any commercial or financial relationships that could be construed as a potential conflict of interest.

## Publisher’s note

All claims expressed in this article are solely those of the authors and do not necessarily represent those of their affiliated organizations, or those of the publisher, the editors and the reviewers. Any product that may be evaluated in this article, or claim that may be made by its manufacturer, is not guaranteed or endorsed by the publisher.
