# Supplementary figures and images for: Long-term outcomes of COVID-19 intensive care unit survivors and their family members: a one year follow-up prospective study
Source: Front Public Health. 2023 Aug 8;11:1236990. doi: 10.3389/fpubh.2023.1236990 (PMC10442651; doi:10.3389/fpubh.2023.1236990)

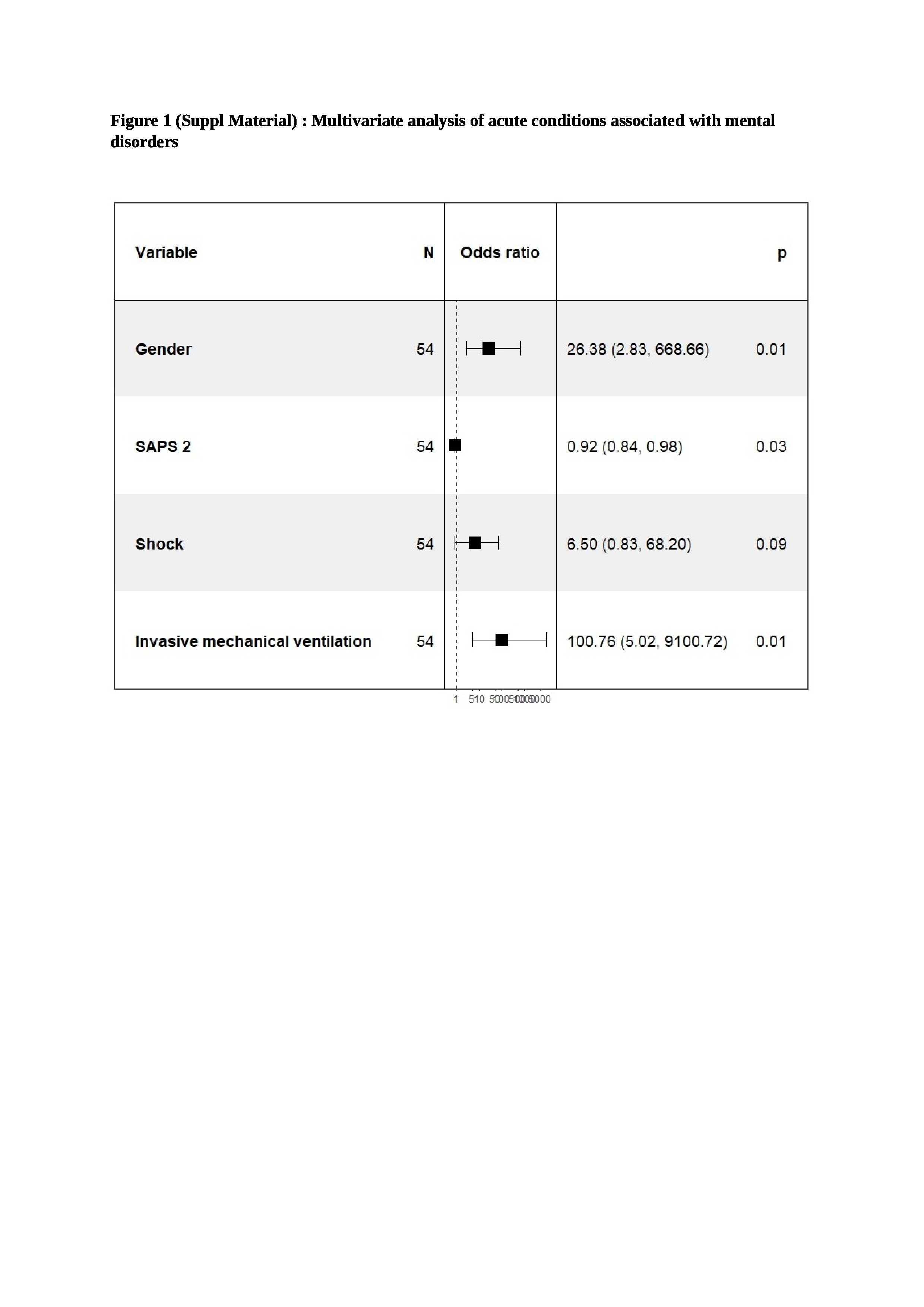

Supplement: Supplementary file 1 [file Image_1.JPEG]

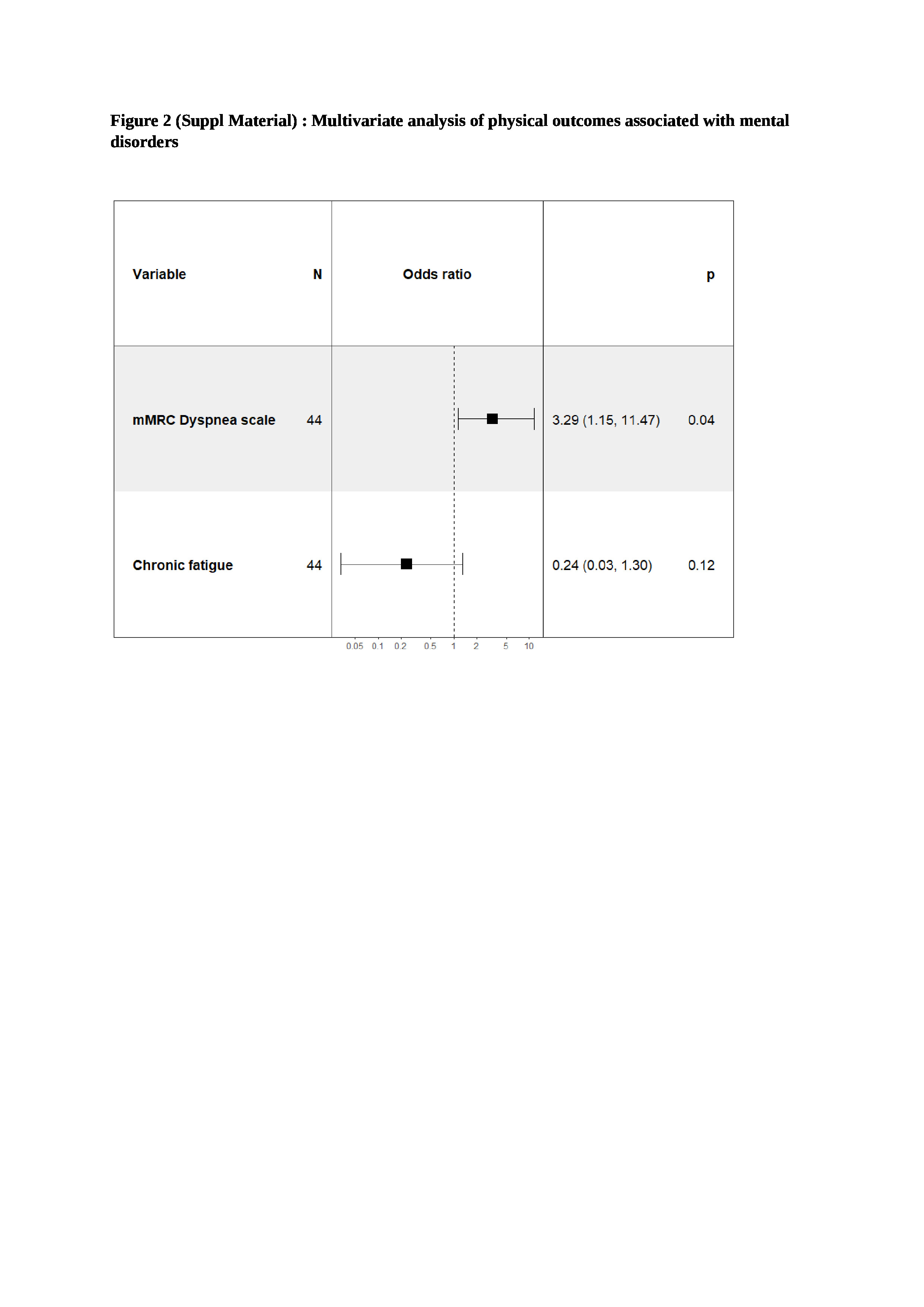

Supplement: Supplementary file 2 [file Image_2.JPEG]
